# *From supportive care to trauma*: training integrative practitioners in the treatment of acute stress disorder

**DOI:** 10.1007/s00520-024-09090-1

**Published:** 2024-12-21

**Authors:** Eran Ben-Arye, Dori Rubinstein, Yael Keshet, Noah Samuels, Gali Stoffman, Mooli Lahad, Alon Reshef, Elad Schiff

**Affiliations:** 1 Ruth and Bruch Rappaport Faculty of Medicine, Technion-Israel Institute of Technology, Haifa, Israel; 2 Integrative Oncology Program, The Oncology Service, Lin, Carmel, and Zebulun Medical Centers, Clalit Health Services, 35 Rothschild St., Haifa, Israel; 3 Community Stress Prevention Center, Kiryat Shmona, Israel; 4 Department of Sociology, Western Galilee College, Acre, Israel; 5 Center for Integrative Complementary Medicine, Shaare Zedek Medical Center, Faculty of Medicine, Hebrew University of Jerusalem, Jerusalem, Israel; 6 Integrative Medicine Service, Barzilai University Medical Center, Ashkelon, Israel; 7 International Academic Affairs Unit, Tel Hai College, Kiryat Shmona, Israel; 8 Department of Psychiatry, Ha’Emek Medical Center, Afula, Israel; 9 Chairperson, The Society of Complementary Medicine, Israel Medical Association, Department of Internal Medicine & Integrative Medicine Service, Bnai-Zion Medical Center, Haifa, Israel

**Keywords:** Acute stress disorder, Integrative medicine, Pain, Narrative-based medicine, Acupuncture, Supportive care, Medical education

## Abstract

**Objective:**

The present conflict in Israel has led to a surge in cases of acute stress disorder (ASD). The study examined a training program for integrative medicine (IM) providers working in supportive and palliative care settings, teaching clinical skills for treating ASD.

**Methods:**

A 10-h online training program, designed by supportive care trained IM and mental health professionals was attended by a group of 32 IM providers. The impact of the course was assessed using pre- and post-training questionnaires, which underwent qualitative evaluation. Three open-ended questions addressed expectations from the program, anticipated barriers to combining IM with mental health interventions, and explored willingness for multi-disciplinary collaboration. A conventional content analysis was used, where coding categories are derived directly from the text data. Narratives were analyzed using ATLAS.ti software for systematic coding.

**Results:**

Narrative themes identified within the group of 32 trainees included expectations regarding facilitating a multi-disciplinary integrative model of care, enriching the ASD-related clinical “toolbox,” increasing the effectiveness of IM treatments, and reducing IM treatment-associated risks. Insights were provided for bridging communication gaps between IM practitioners and mental health providers, supporting the multi-disciplinary collaboration.

**Conclusions:**

ASD-focused training for IM practitioners may increase their level of clinical skills and advance collaboration with mental health providers. Future research examining the feasibility of the integrative model and its implementation in supportive care setting is warranted.

## Introduction

Services providing complementary and integrative medicine (IM) are increasingly becoming part of supportive and palliative care settings. In the oncology setting, this is based on clinical practice guidelines supporting the effectiveness and safety of these therapies for the treatment of pain, anxiety, depression, anxiety, and fatigue [1–3]. In contrast, integrative models in mental health are far less prevalent, with the exception of pioneering mental health collaborative initiatives in the Netherlands, the USA, and Israel [4–6].

Current research on the effectiveness and safety of IM for acute stress disorder (ASD) and post-traumatic stress disorder (PTSD) is limited. In an editorial on the international role of CIM for PTSD, Niles et al. addressed non-specific and specific outcomes, particularly with mind–body therapies (e.g., meditation, relaxation), while suggesting a therapeutic potential in arousal reduction, emotion regulation, and posttraumatic growth [7]. In their systematic review on the treatment of PTSD-related symptoms, Zhu et al. found a significant benefit in the reduction of depression and anxiety with mind–body exercises [8]. Randomized controlled trials (RCTs) have shown IM to be beneficial in the treatment of PTSD, including acupuncture [9] and ear acupuncture for sleep disturbances [10] and Yoga for depression, anxiety, intrusion, and avoidance in female survivors of motor vehicle accidents [11], with a meta-analysis of four RCTs on mindfulness-based therapies for stress reduction [12].

The current conflict in Israel has led to an increase in cases of ASD, with the exposure of an overwhelming number of civilians to physical and emotional trauma, as well as displacement of entire communities from towns, villages, and kibbutzim to safer areas in the country. The present study examined an ASD-focused training program for IM practitioners, addressing the acute need of a national magnitude to reduce psychological morbidity, while recognizing the limited availability of trauma-trained mental health providers, and the response of IM practitioners across the country who have been volunteering to provide relief, despite their lack of training in trauma care, especially for ASD.

## Methods

### Study setting and participants

The training program took place online, entailing 10 h of lectures and experiential training. IM practitioner trainees (acupuncturists and touch therapists) were working in integrative settings of supportive and palliative care, mainly in northern Israel.

### Goals of the training program

The primary goal of the training program was to provide IM practitioners with a knowledge-based approach and clinical skills for treating hospital personnel and patients presenting with ASD-related symptoms, most significantly the arousal symptom cluster (e.g., sleep problems, irritability, rage attacks, highly and abnormally alert to surroundings, distractibility, unusually strong reflexive reaction to a sudden event in the environment) as defined in the DSM-V [13]. The program exposed trainees to both IM and mental health therapeutic approaches, providing diagnostic and therapeutic skills concerning ASD-related symptoms. Secondary goals of the training program included facilitating a bi-directional communication and referral process between IM practitioners and mental health providers treating patients diagnosed with ASD, within an integrative multidisciplinary model of care.

### Design of the training program

The training program syllabus was co-designed and mentored by three board members of the Society for Complementary Medicine, Israel Medical Association (EBA, ES, GS), and two psychologists with ASD and PTSD expertise from the Community Stress Prevention Center, Kiryat Shmona, Israel (DR and ML). The design process for the program was based on the following considerations:

- Acknowledgements by the mental health mentors of the limited effectiveness of current psychotherapeutic approaches in preventing and alleviating specific ASD-related symptoms. Based on extensive clinical experience, it was hypothesized that the addition of IM treatments could provide an additional clinical benefit, mainly in alleviating arousal symptoms, with the hope of either preventing the development of PTSD or at least reducing its severity.
- Experience of the IM mentors in addressing quality of life-related concerns in both inpatient and outpatient CIM settings (e.g., oncology, palliative care, internal medicine, emergency medicine, and surgery) for the relief of pain, anxiety, shortness of breath, and gastro-intestinal concerns.
- Experience of both groups of mentors in working with multi-disciplinary teams of healthcare providers, bridging communication gaps between professionals with different experiences and training, thereby increasing the effectiveness of the therapeutic process.
- Recognition of the urgency of the program, in light of the current conflict in southern Israel. The syllabus was designed with the goal of implementing the knowledge and skills acquired within a short period of time, with the assumption that the conflict could continue and even expand to the north of the country.

The design of the syllabus began with a search of the scientific literature (PubMed/Medline) for evidence (for and against) the effectiveness and safety of CIM treatments for ASD and PTSD. The following key words were used for this purpose: ASD, PTSD, patient/caregiver, expectations, concerns, communication, integrative medicine, complementary medicine, acupuncture, traditional Chinese medicine, reflexology, touch therapies, and Anthroposophic medicine. A preliminary draft of the syllabus was presented to two non-MD CIM practitioners: one integrative physician trained in traditional Chinese medicine (TCM) and Anthroposophic medicine, one art therapist, and one social worker trained in mind–body therapy. After receiving feedback from all participants in the design process, a final version of the syllabus was prepared.

### Core themes of the ASD integrative training syllabus

Table 1 presents core themes of the integrative ASD training syllabus addressing the following themes:

1. *Introduction to ASD and PTSD:* Trainees were taught how to diagnose and assess ASD-related symptoms, using tools such as the National Stressful Events Survey Acute Stress Disorder Short Scale, NSESSS [14], and to administer the Measure Yourself Concerns and Wellbeing (MYCAW) questionnaire, a tool used in a wide range of clinical integrative medicine settings [15].
2. *Introduction of the CIM approach to mental trauma.* CIM practitioners were divided into those trained in acupuncture and TCM and those in touch therapy (mainly reflexologists). Emphasis was placed on the CIM approach to the treatment of pain, anxiety, and ASD-related symptom clusters.
3. *Communication, referral, and clinical collaboration between CIM and mental health providers.* This theme focused on identifying barriers and facilitators of the multidisciplinary CIM and mental health teams, while highlighting aspects related to the effectiveness (e.g., expanding the “toolbox” of therapeutic options) and safety (e.g., relative and absolute contra-indications) of CIM therapies, and when to refer patients to a mental health provider, etc.
4. *Preventing secondary traumatization of CIM practitioners*. Providers were educated about the risk for secondary traumatization resulting from their exposure to the patient’s experience.

**Table 1:** Core themes of the acute stress disorder (ASD) integrative training syllabus

Theme	Specific highlights
Introduction to ASD and PTSD	Trainees were taught how to diagnose and assess ASD-related symptoms, using tools such as the National Stressful Events Survey Acute Stress Disorder Short Scale, NSESSS^1^, and to administer the Measure Yourself Concerns and Wellbeing (MYCAW)^2^ questionnaire, a tool used in a variety of integrative medicine clinical settings
Introduction to the IM approach to mental trauma	Introduction to the IM approach to mental trauma. IM practitioners were divided into those trained in acupuncture and TCM and those in touch therapy (mainly reflexologists). Emphasis was placed on the IM approach to the treatment of pain, anxiety and ASD-related symptom clusters
Communication, referral process and clinical collaboration	This theme focused on identifying barriers and facilitators of the multidisciplinary IM and mental health teams, while highlighting aspects related to the effectiveness (e.g., expanding the “toolbox” of therapeutic options) and safety (e.g., relative and absolute contra-indications) of IM therapies, and indications for referral of patients to a mental health provider, etc
Preventing secondary traumatization of IM practitioners	Providers were educated regarding the risk for secondary traumatization resulting from their exposure to the patient’s experience

*ASD*, acute stress disorder; *PTSD*, post-traumatic stress disorder; *IM*, integrative medicine

^1^Efendi GY, Temeltürk RD, Çakmak IB, Dinçer M. Surviving the immediate aftermath of a disaster: a preliminary investigation of adolescents’ acute stress reactions and mental health needs after the 2023 Turkey earthquakes. Children (Basel). 2023;10(9):1485

^2^Paterson C, Thomas K, Manasse A, Cooke H, Peace G. Measure Yourself Concerns and Wellbeing (MYCaW): an individualised questionnaire for evaluating outcome in cancer support care that includes complementary therapies. Complement Ther Med. 2007;15(1):38–45

### Study questionnaire

The study questionnaire was developed by the authors and was based on tools published in medical education studies of healthcare providers undergoing IM training [16, 17]. The questionnaire design was enriched by the diverse multi-disciplinary background of participating IM-trained physicians, psychologists, and a sociologist. The questionnaire was administered anonymously, beginning with items addressing demography (age, gender, country of origin, religion, religiosity) and professional status (academic degree, mental and CIM care training, years of practice). Qualitative study outcomes were derived from three open-ended questions addressing expectations from the course, anticipated barriers to combining mental health and IM therapies, and their willingness to collaborate within a multi-disciplinary team. Questionnaires were completed prior to and at the end of the training program, with follow-up assessment three weeks after course completion. Validation of the study questionnaire was not feasible due to the urgency of war-related circumstances, nor were structured role play simulations or case studies included, for this reason as well.

### Qualitative analysis

A conventional content analysis was used for trainees’ replies to the open-ended questions in the questionnaire at pre-, post-, and follow-up evaluations. Coding categories were derived directly from the text data (inductive category development) [18]. The content analysis was conducted by a medical sociologist (YK) using the ATLAS.ti version 8 textual data analysis software (ATLAS.ti Scientific Software Development, Berlin, Germany).

### Ethical considerations

The study protocol was approved by the Ethics Review Board (ERB, Helsinki Committee) at the Barzilai University Medical Center (0093–23-BRZ), Ashkelon, Israel, and exempted from requiring IRB approval at the Carmel Medical Center in Haifa, Israel. The training program evaluation was part of a larger study initiated at the beginning of the current conflict in Israel on October 7, 2023, with the goal of assessing the impact of IM interventions on anxiety and wellbeing among healthcare providers exposed directly or indirectly to terror, trauma, or war.

## Results

### Description of the study group

A total of 32 IM practitioners participated in the training program, majority are female (21, 66%), Israeli-born (27, 84%), married or living with a partner (23, 72%), and with a mean age of 50.06 ± 7.8 years. All participants were Jewish, most identifying as secular (28, 87.5%) with a spiritual quest score of 5.5 ± 1.9 points (median 6) on a 1- to 7-point scale. The majority were trained in TCM (24, 75%) and had been working in IM for a mean number of 18.1 ± 7.7 (standard deviation) years, 14 (44%) had academic degrees (mainly MDs and RNs), and only 3 (9%) had mental health training.

**Fig. 1:**
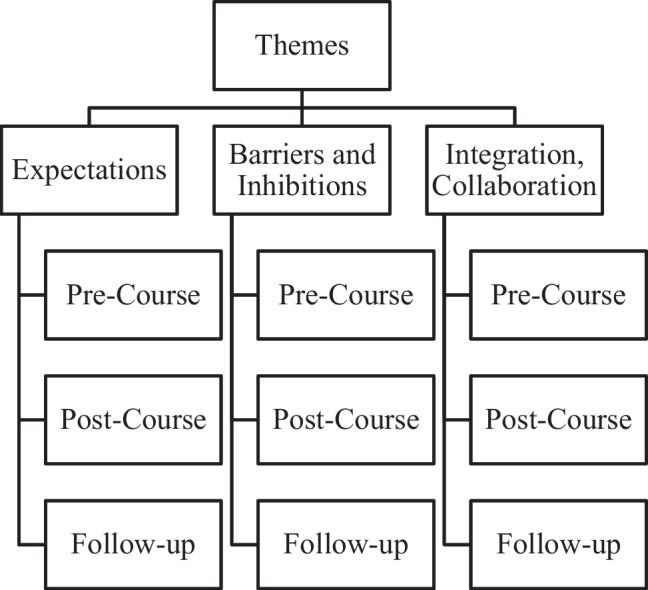
Key themes identified in the qualitative assessment

Of the 32 trainees, 25 responded to pre- or immediate post-training questionnaires. A 3-week follow-up assessment was available for 29 respondents, of which 23 attended online post-course lectures, with 21 treating patients and/or healthcare providers expressing ASD-related concerns following the course. Three key themes for short pre- and post-course and 3-week follow-up narratives are presented in Fig. 1. Table 2 presents the three central themes highlighted in this qualitative analysis.

**Table 2:** Qualitative analysis emerging themes and narratives

Theme	Selected trainees’ narratives
Trainees’ expectations	“How to diagnose and identify patients who are in a state that requires health care professionals from the field of trauma, and to refer to them…to know when, and if appropriate… when not to ask questions which are too deep, or invasive.” (a 33-year-old female TCM therapist) “The training program allowed me to “load up” with my first dose of knowledge, as well as a new approach to stress-related disorders. Following the course, we were able to acquire additional knowledge during the supplementary “Zoom” meetings, and most significantly were able to implement (these) concepts in their meetings with patient and medical team.” (a 59-year-old male integrative physician)
Barriers and inhibitions	“I am afraid that I won’t know when I can’t take any more. My lack of experience and knowledge in this field…my strong desire to help, and quickly…my inexperience working with a team of experts, with events on this scale and extremity, as we are experiencing now…” (a 45-year-old female TCM practitioner) “After the training, I felt much more confident. My only concern was that ‘moving the Chi’ would bring the memories (i.e., of the trauma) to the surface…in an uncontrolled manner…” (a 52-year-old female TCM practitioner)
Integration and collaboration	“Of course, we need to work with them with excellent collaboration. We were not trained to provide emotional and medical relief, and we have to have their professional support.” (a 45-year-old TCM practitioner) “The most essential of the challenges is creating a common language, or jargon; reaching common therapeutic expectations from both sides; and a true willingness to collaborate.” (a 41-year-old male TCM practitioner) “The added value, as far as I’m concerned, is providing the most effective and comprehensive treatment to the patient, who will benefit from all therapeutic approaches for what is most appropriate for him.” (a 51-year-old female TCM and Shiatsu therapist) “From the therapist working with those displaced due to the current conflict, I see that it is good that emotional care be provided following touch and acupuncture treatments… or even provided together…for those who can talk, and for those who can’t…” (a 66-year-old female nurse who combines acupuncture with reflexology in her practice) “I believe in integration. The added value is the expanded ‘tool box’… and perceiving the patient in multiple ways. There is room for brainstorming, for thinking together with other health care professionals, and a place for better mutual productivity. The challenges facing the inter-disciplinary work setting are similar to those faced wherever there a varying attitudes and approaches. This requires patience, inclusion, listening, compromising, foregoing of ego and creating a guiding protocol.” (a 55-year-old female TCM practitioner)

### Theme I: Trainees’ expectations

The majority of pre-training expectations addressed in the narratives was focused on interactions with patients, including learning how to approach those suffering from ASD, avoiding potential risks (“what to do, and what not to do…how I can make things worse, and what to avoid”), creating a new therapeutic “toolbox,” and developing new and improved therapeutic and communication skills, including with HCPs from another discipline.

Some respondents related to their role as therapists, as opposed to their “doing” role focusing on the needs of the patient. This included concerns regarding secondary traumatization from the patient’s description of their traumatic experience (“…how to protect myself, without getting enmeshed…”), as well as getting support and mentoring.

At the end of the course, narratives became more focused on training outcomes, with many participants expressing satisfaction from the fact that the process met their expectations vis-à-vis knowledge, insights, and adopting a systematic approach integrated within a psycho-educational perspective. At the same time, many respondents felt that the training was too short and that a more in-depth learning process was required, particularly through roleplay simulations, case studies, and discussions within a multi-disciplinary team setting.

At the 3-week follow-up assessment, the majority of respondents (21 of 29) felt that their expectations had been met and that they had gained more confidence in treating patients exposed to trauma. Several respondents pointed out the potential risks associated with IM treatment of ASD and related symptoms without sufficiently relevant training:

Recognizing the importance of the subject, of this unique therapy…of the harm that might be caused to these patients without treatment, or with improper treatment…. (a 50-year-old female TCM practitioner).

### Theme II: Barriers and inhibitions

Barriers and inhibitions to including IM in the treatment of ASD were reported by the majority of trainees at baseline (23/32, 72%), less so at the end of training (12/25, 48%), and at the 3-week follow-up assessment (11/29, 38%). Pre-training concerns included the risk of causing harm due to lack of mental health training, lack of professionalism, difficulty in distinguishing between effective and risky therapeutic outcomes (“fear that I won’t know if I’m causing more harm than good…”), and inability to handle non-desirable effects of the treatment (“…that the treatment will awaken a response that I won’t be able to encompass”).

The combination of an intense commitment to the therapeutic process, along with a fear of causing harm to both the patient and therapist through secondary traumatization (“I know that at that moment I will absorb everything, but am afraid of the long-term effects…”), was often followed by a call for further mentoring by a mental health provider.

Baseline reservations were also reported at the end of training and at the 3-week follow-up assessment (“My concern is my inability to take it in…maybe also to bring it to the surface…”). However, these concerns were expressed less frequently and with less intensity post-course, with a more developed sense of self-confidence. Coping with patients and traumatic narratives was perceived as a potentially overwhelming experience (“how can I ‘cleanse’ these feelings, so that I can be effective over the long term…”), while continuously being part of a multi-disciplinary collaboration:

The fear of being alone when facing very difficult emotional situations. I hope that a mental healthcare provider will be there throughout the consultation… (a 60-year-old female integrative physician, working in TCM and Anthroposophic medicine).

### Theme III: Integration and collaboration

A high level of expectation was found among the majority of trainees at all stages of the study assessment regarding the multi-disciplinary collaboration between IM and mental health providers. Pre-training narratives were supportive of integration within the context of the therapeutic “toolbox” (“…the tools that each one brings from their own world”), enhancing the effectiveness of the treatment and safety and enhancing patient-centered care, with feedback and support. The absence of mental health trainees participating in the course was also mentioned.

At post-training, trainees’ narratives highlighted the importance of collaboration, using terms attributed to a synergy between teams (“the primary challenge is learning to work together, so that one plus one is much more than just two…”), facilitation, and holism.

At the 3-week follow-up assessment, integrative skills were being implemented in trainees’ clinical work, with insights into the benefits of the integrative multi-disciplinary approach. While highly supportive of the integrative process, participants also expressed their concerns regarding the barriers to the multi-disciplinary collaboration, suggesting ways to bridge gaps between the two approaches to patient care.

## Discussion

The present study examined the impact of a training program whose goal was to enable IM practitioners working in supportive care settings to help patients suffering from ASD and related symptoms in response to the current conflict in Israel. The study setting provides a unique opportunity to explore an integrative mental health education model, designed by a collaborative team of IM-trained physicians and mental health providers. Qualitative analysis identified themes which included expectations on how to facilitate a multi-disciplinary integrative model of care, enriching the available ASD-related clinical “toolbox,” enhancing the effectiveness of the therapeutic process, and reducing treatment-associated risks.

The design of the training program was extremely challenging, especially the rapidity in which the syllabus was created in order to meet the acute and overwhelming need due to the current conflict in Israel. While most IM practitioners in the country lack formal training in mental health disciplines, other such projects have been successful in the integration of these practices in other models of care in Israel, such as oncology, palliative care, and obstetrics [19].

The study findings are important in light of the discussion on the model of interdisciplinary communication and collaboration between IM and mental health providers [20]. While acknowledging the existence of inter-disciplinary barriers, as well as a lack of mental health proficiency, trainees were able to provide profound reflections on how to promote collaboration and integration, sharing their expectations from the process. At the same time, they were able to describe what they felt was the added clinical value of IM to ASD treatment programs, addressing symptoms which are not optimally treated by mental health providers. In the multi-disciplinary integrative model of care, the acupuncturist and reflexologist may be able to help reduce arousal symptoms, while referring patients with suspected ASD-related “red flags” (e.g., dissociation, flashbacks, suicidal ideation) to the mental health provider. The referral process may be considered bi-directional, initiated by the mental health provider to the IO practitioner, including when a non-verbal therapy is sought (e.g., touch therapy purposed to encourage “grounding” in a patient highly alert to surroundings). Trainee narratives suggest that integration is not only “working together” or “not being alone,” but also ensuring safety and effectiveness, particularly with respect to challenging symptoms for which one therapeutic approach is not effective enough.

The present study has a number of methodological limitations that need to be addressed in future research. These include the small sample size, lack of study questionnaire validation, and the fact that only a selected group of IM practitioners, mainly those working already in integrative settings of care, were included. The lack of perspective diversity through the selection of a largely experienced IM workforce with a similar cultural background is also an important point to note. This is a major selection bias, particularly when exploring the trainees’ perception of integration in ASD care. Another significant limitation is the absence of mental health providers as trainees. The data provided through short narratives appears to have reached thematic saturation, and the study results may have limited transferability when taking into consideration similar contexts of care and should be further explored in other Israeli and international trauma settings. Such research should consider the inclusion of trainees with diverse professional backgrounds, including psychologists, psychiatrists, social workers, and art therapists, in addition to IM-trained practitioners. It should also explore whether gender and trainees’ culture and religion play a role in the training process, especially when these factors are more diverse. Finally, a follow-up assessment should be considered in order to explore the implementation of the training outcomes in real-life clinical practice.

In conclusion, the present study suggests that the majority of IM trainees undergoing an ASD training program during war-related conflict report increased levels of clinical skills which may promote collaboration with mental health providers. More studies are needed to explore the feasibility of similar training programs, with the inclusion of mental health and supportive care providers, examining both the impact of the course and its implementation in clinical ASD practice.

## Data Availability

No datasets were generated or analysed during the current study.

## Code availability

N/A.
